# Prediction of the Post-Pubertal Mandibular Length and Y Axis of Growth by Using Various Machine Learning Techniques: A Retrospective Longitudinal Study

**DOI:** 10.3390/diagnostics13091553

**Published:** 2023-04-26

**Authors:** Tyler Wood, Justina O. Anigbo, George Eckert, Kelton T. Stewart, Mehmet Murat Dundar, Hakan Turkkahraman

**Affiliations:** 1 Department of Orthodontics and Oral Facial Genetics, Indiana University School of Dentistry, Indiana University Purdue University at Indianapolis, Indianapolis, IN 46202, USA; woodt@iu.edu (T.W.);; 2 Department of Biostatistics and Health Data Science, Indiana University School of Medicine, Indianapolis, IN 46202, USA; 3 Department of Computer & Information Science, Indiana University Purdue University at Indianapolis School of Science, Indianapolis, IN 46202, USA

**Keywords:** artificial intelligence, machine learning, growth and development, mandible

## Abstract

The aim was to predict the post-pubertal mandibular length and Y axis of growth in males by using various machine learning (ML) techniques. Cephalometric data obtained from 163 males with Class I Angle malocclusion, were used to train various ML algorithms. Analysis of variances (ANOVA) was used to compare the differences between predicted and actual measurements among methods and between time points. All the algorithms revealed an accuracy range from 95.80% to 97.64% while predicting post-pubertal mandibular length. When predicting the Y axis of growth, accuracies ranged from 96.60% to 98.34%. There was no significant interaction between methods and time points used for predicting the mandibular length (*p* = 0.235) and Y axis of growth (*p* = 0.549). All tested ML algorithms accurately predicted the post-pubertal mandibular length and Y axis of growth. The best predictors for the mandibular length were mandibular and maxillary lengths, and lower face height, while they were Y axis of growth, lower face height, and mandibular plane angle for the post-pubertal Y axis of growth. No significant difference was found among the accuracies of the techniques, except the least squares method had a significantly larger error than all others in predicting the Y axis of growth.

## 1. Introduction

Craniofacial growth is often described in magnitude, direction, and velocity. Within the craniofacial complex, the mandible is the skeletal component with the greatest potential for growth [1]. This fact plays a significant role in the field of orthodontics and in the management of skeletal malocclusions. Sometimes, the growth magnitude and direction can favor the orthodontist’s goals. In patients with Class II tendencies, forward growth of the mandible can aid the orthodontist in the correction of the malocclusion. Conversely, the growth of the mandible can also be a drawback to treatment. In Class III patients, mandibular growth becomes a large problem, significantly adding to case difficulty for the practitioner and potential stress to the patient. Baumrind et al. [2] noted that mandibular growth can also be a substantially negative factor in Class II patients if the patient is considered a “backward grower” or in that their mandibles tend to have a rotation pattern inferior and posteriorly.

As early as the 1950’s, Björk [3] studied the growth patterns of the face by placing metallic implants in the jaws of growing children to use as fixed reference points and monitoring them with radiographs for several years. He sought to understand the normal variation of growth in children to assess growth trends at an early age. His study was pivotal in understanding that mandibular growth stems from the condyle, which displaces the mandible downward and forward creating a generally forward rotation of the mandible. Building on Björk’s implant studies, Skieller et al. [4] continued to investigate the amount and direction of growth in the mandible and found that the mandible grew an average of 6° forward over a 6-year time interval. They also showed that 86% of the variability of mandibular rotation between subjects was a result of four different variables: (1) mandibular inclination; (2) intermolar angle (MOLs-MOLi); (3) shape of lower border (ML&MLP); and (4) inclination of symphysis (CTL-NSL). However, when Leslie et al. [5] repeated the methodology by tracing the same lines and using the same equations, they attained highly varying results, proving the previous study unreliable. They also performed a Monte Carlo study which mirrored the Skieller analysis but used random numbers instead of actual cephalometric data and found a mean of 84% and median of 94% of mandibular growth variability explained by meaningless data.

More recently, mathematical models have been developed for predicting the amount of mandibular growth in children. Although these proved promising, they do not account for individual variability [6]. Some mathematical models proved slightly more accurate in predicting direction more than amount of mandibular growth, but these models still had room for improvement [7]. A mathematical model established by Buschang et al. [6] compared mean annual velocities of growth to a mathematical model based on a population’s growth curve. This model used multilevel model statistics to summarize mean growth curves and variations between measurement occurrences and between subjects. Their study reflected 76–77% accuracy for predicting growth for males and females, respectively. They noted bias caused by expected variations in growth that methods of prediction could not account for. The study by Oueis et al. [7] sought to predict the mandibular growth of Japanese children between ages 4 and 9. Using cephalometric tracings and multiple linear regression analysis, several mathematical equations were developed for growth prediction. The regression analysis used in the study explained 72% of variability of mandibular growth direction and 61% of growth amount. Although accuracy of these studies in predicting growth amount were not very high, these studies nonetheless demonstrated the importance of statistical and mathematical analysis in predicting mandibular growth.

Humans are limited by time and in capability to process the amount of information necessary to predict both direction and amount of mandibular growth with both validity and reliability. Recent advances in artificial intelligence (AI) have made it possible to analyze big data in a very short period of time, and to solve previously unsolved problems through supervised, unsupervised, or reinforcement learning. In most basic terms, AI describes computers mimicking human intelligence using extensive data from past examples of similar behavior.

Previous applications of AI in orthodontics, were more focused on automated cephalometric landmark identification [8,9], automated facial analysis [10], evaluation of facial attractiveness [11], classification of craniofacial skeletal patterns [12,13], surgery/non-surgery decision in class III patients [14,15], semantic segmentation of maxillary teeth and palatal rugae in two-dimensional images [16], auto-segmentation of the maxilla in cone beam computerized tomography images [17], fully automated determination of the cervical vertebrae maturation stages [18], dental and skeletal age assessments [19,20], and orthodontic treatment planning [21,22,23,24,25,26]. The authors of this study asked whether the power of supervised ML methods could be harnessed for a deeper understanding of the underlying mechanisms of pubertal mandibular growth by using data from human growth studies. The American Association of Orthodontists Foundation (AAOF) Craniofacial Legacy Collection made the desire to test this question a reality by sharing invaluable sources of information from previous growth studies. In an early attempt, Jiwa et al. [27] used a novel deep learning algorithm to predict X and Y coordinates of 17 mandibular landmark predictions on selected serial cephalograms of 101 growing subjects and compared the accuracy of the methods with Ricketts’s growth prediction. However, their algorithm was not deemed to be accurate for generalized 2-year growth prediction. None of the 12 skeletal landmarks or 5 dental landmarks had a prediction error below the clinical reference mean of 1.5 mm. They suggested an increase in data volume and training to improve prediction accuracy.

To our knowledge, no other studies have used ML techniques to analyze longitudinal craniofacial cephalometric input data in predicting the post-pubertal mandibular length and Y axis of growth in males. Therefore, the aim of this study was to create algorithms by using various ML techniques in order to predict the post-pubertal mandibular length and Y axis of growth in males and to compare their accuracies. The null hypotheses to be tested were that there would be no difference between the predicted and actual values of post-pubertal mandibular length and Y axis of growth, and there would be no difference between the accuracies of the algorithms.

## 2. Materials and Methods

This study was approved as a non-human subjects research (NHSR) project by the Institutional Review Board (IRB) of Indiana University (Protocol #11487).

### 2.1. Study Sample

The data for this retrospective study consisted of digital cephalometric radiographs acquired from the AAOF Craniofacial Legacy Collection, which houses images from subjects from the Bolton Brush Growth, Burlington Growth, Denver Growth, Fels Longitudinal, Forsyth Twin, Iowa Growth, Matthews Growth, Michigan Growth, and Oregon Growth studies. Inclusion criteria included male subjects with cephalograms at specific timepoints, T1 representing the pre-pubertal stage (Mean age ± SD: 11.85 ± 0.46 yrs), T2 representing the pubertal stage (Mean age ± SD: 13.82 ± 0.49 yrs), T3 representing the post-pubertal stage (Mean age ± SD: 15.80 ± 0.57 yrs). Subjects were also determined to be Angle’s Class I in the database. Subjects with craniofacial anomalies, apparent skeletal asymmetries, and missing timepoints of interest, or cephalograms that were of poor quality were excluded from the study. A total of 163 cases met the inclusion criteria and were selected for use in this study.

### 2.2. Sample Size Justification

The study used cephalometric radiographs of 163 samples obtained at 3 timepoints. Of these, 70% (114) were used for the training set and 30% (49) were used for the testing set. With 49 samples in the testing set, the 95% confidence interval for the intraclass correlation coefficient (ICC) has a width of 0.21, extending from 0.67 to 0.88, if the ICC is 0.80; higher ICCs have shorter confidence interval widths.

### 2.3. Data Collection

Images were transferred from the AAOF repository and uploaded into Dolphin Imaging V. 11.95 (Dolphin Imaging and Management Solutions, Chatsworth, CA, USA). Thirty-six hard tissue landmarks were plotted by a single investigator (T.W.) on each image and were used to calculate 39 linear and angular measurements, displayed in Table S1. Measurements were calibrated by using Dots Per Inch (DPI) as provided by the AAOF. When magnification errors were detected, images were printed at 1:1 scale and ruler length measured for accuracy and then the digital ruler was used to calibrate measurements for those images. Demographic and cephalometric data were then transferred into a spreadsheet, and stored in a secure cloud service (OneDrive, Microsoft Co., Redmond, WA, USA). A research randomizer was used to randomly select 20 images to retrace for intra-examiner repeatability assessment. The ICCs were used to assess repeatability of the measurements.

### 2.4. Algorithm Training and Testing

**Figure 1:**
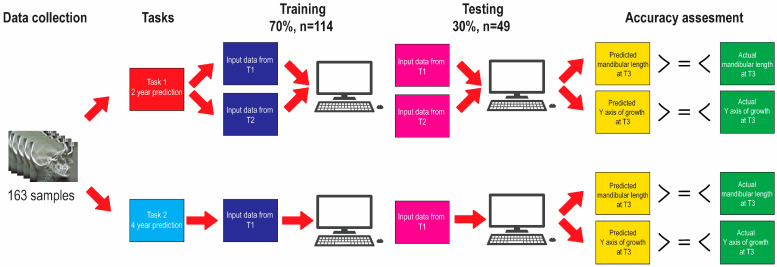
Algorithm training and testing pipeline.

The algorithm training and testing pipeline is illustrated in Figure 1. Data were randomly distributed into two datasets. The training set consisted of 70% of the samples’ data (114 samples) and the remaining 30% (49 samples) were allocated for the testing. The training set was used for training the supervised machine learning models. In this step, the dependent variables mandibular length and Y axis of growth at T3 were given along with the 39 linear and angular independent variables at T1 and T2. Trained models were then evaluated on the testing set in terms of their accuracy in predicting dependent variables mandibular length and Y axis of growth at T3. The prediction task was repeated twice; first using predictors from T1 and T2 together, and then with predictors from T1 alone.

Seven regression algorithms were used for the analyses: least squares (linear), ridge, lasso, elastic net, XGBoost, random forest, and a neural network. All experiments were performed in Spyder 4.1.5 using Python 3.7.9. (Python Software Foundation, Fredricksburgh, VA, USA). For least squares, ridge, lasso, elastic net, and random forest, the sklearn package version 1.0.2 (NumFOCUS, Austin, TX, USA) was used. For XGBoost, the xgboost package version 1.5.0 (DMLC, USA) was used. For neural net, the Keras package version 2.4.0 (Keras, Mountain View, CA, USA) was used in the TensorFlow version 2.4.3 (Keras, Mountainview, CA, USA) platform.

### 2.5. Statistical Analyses

The root mean square error (RMSE), mean absolute error (MAE), mean error (ME), and ICCs were calculated for each technique to evaluate the agreement between the predicted and actual outcome measurements. The accuracy percentage of the methods were calculated by the formula (1 − (MAE/Actual value) × 100). Analysis of variances (ANOVA) was used to compare methods and between time points used for predictors for differences between predicted and actual measurements at T3. The ANOVA included fixed effects for method, time points used for predictors, and their interaction, and random effects for patient and interactions of patient with method and time points used for predictors. Two-sided 5% significance levels were used for all tests. All analyses were performed using SAS version 9.4 (SAS Institute, Inc., Cary, NC, USA).

## 3. Results

### 3.1. Reliability Analysis

The results of the reliability analysis are given in Table S2. Most variables showed excellent repeatability (ICCs > 0.9), with the remainder having good repeatability (0.75 < ICC < 0.9) [28]. The only exception to this was the Holdaway ratio, which demonstrated a reliability of 0.61.

### 3.2. Descriptive Statistics

The descriptive statistics, including mean, standard deviation, minimum and maximum of the cephalometric variables at T1, T2 and T3 are shown in Table S3.

### 3.3. Prediction of the Post-Pubertal Mandibular Length

**Figure 2:**
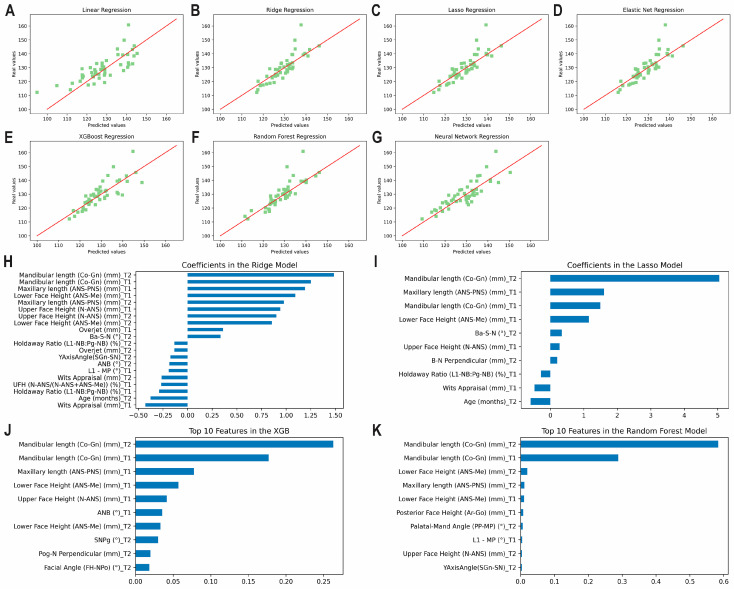
Predicting mandibular length at T3 using predictors from T1 and T2 together. Scatter plots for the predicted and actual values with lines of best fit for the: (**A**) Least Squares (linear); (**B**) Ridge; (**C**) Lasso; (**D**) Elastic Net; (**E**) XGBoost; (**F**) Random Forest; (**G**) Neural Network models. Top correlating coefficients for the (**H**) Ridge model; (**I**) Lasso model; (**J**) XGBoost model; (**K**) Random Forest model.

The results of the prediction analysis of mandibular length at T3 using predictors from T1 and T2, including ME, MAE, RMSE, ICCs and accuracy percentages are given in Table 1 and Figure 2A–G. Accuracy percentages ranged from 95.80% to 97.64% between algorithms employed. All methods demonstrated a good correlation between predicted and actual values (0.75 < ICCs < 0.90). The top correlating coefficients identified by the methods were found to be the mandibular length at T2 followed by the mandibular length at T1, the maxillary length at T1, and lower face height at T1 (Figure 2H–K). Supplemental Figure S1 shows the correlation matrix with heatmap, representing the correlation between the variables used to predict mandibular length at T3 using variables from T1 and T2 combined.

**Figure 3:**
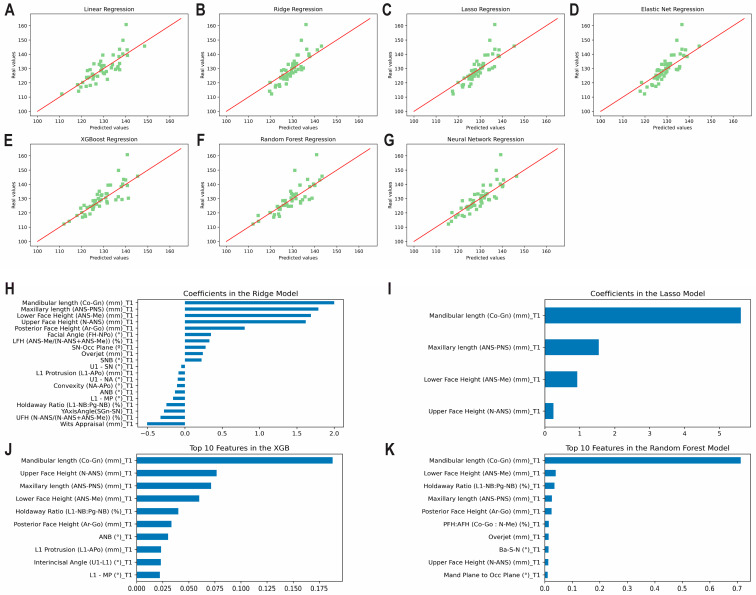
Predicting mandibular length at T3 using predictors from T1 alone. Scatter plots for the predicted and actual values with lines of best fit for the: (**A**) Least Squares (linear); (**B**) Ridge; (**C**) Lasso; (**D**) Elastic Net; (**E**) XGBoost; (**F**) Random Forest; (**G**) Neural Network models. Top correlating coefficients for the (**H**) Ridge model; (**I**) Lasso model; (**J**) XGBoost model; (**K**) Random Forest model.

The results of the prediction analysis of mandibular length at T3 using predictors from T1 alone are shown in Table 1 and Figure 3A–G. Levels of accuracy between 96.94% and 97.33% were observed. All methods revealed a good correlation between predicted and actual values (0.75 < ICCs < 0.90). The top correlating coefficients observed by the methods were found to be the mandibular length at T1 followed by the maxillary length at T1, lower face height at T1, and upper face height at T1 (Figure 3H–K). Supplemental Figure S2 shows the correlation matrix with heatmap, representing the correlation between the variables used to predict mandibular length at T3 using variables from T1 only.

### 3.4. Prediction of the Post-Pubertal Y Axis of Growth

**Figure 4:**
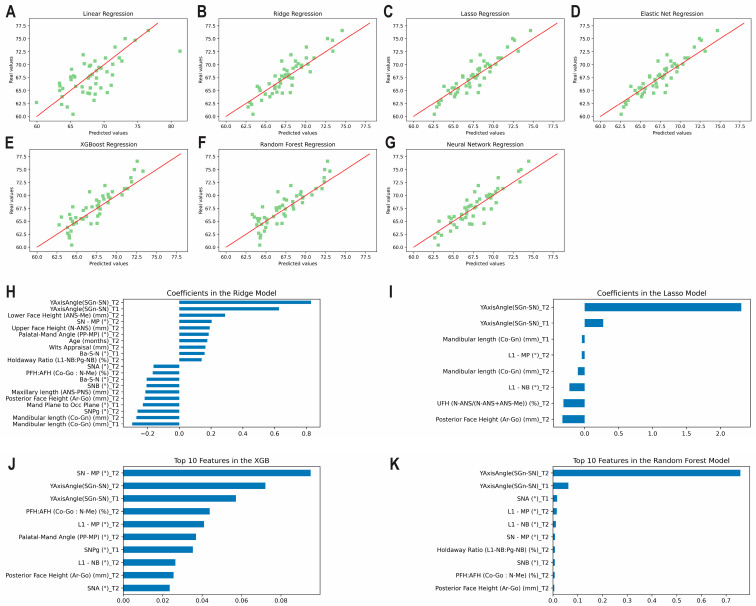
Predicting the Y axis of growth at T3 using predictors from T1 and T2 together. Scatter plots for the predicted and actual values with lines of best fit for the: (**A**) Least Squares (linear); (**B**) Ridge; (**C**) Lasso; (**D**) Elastic Net; (**E**) XGBoost; (**F**) Random Forest; (**G**) Neural Network models. Top correlating coefficients for the (**H**) Ridge model; (**I**) Lasso model; (**J**) XGBoost model; (**K**) Random Forest model.

The results of the prediction analysis of the Y axis of growth at T3 using predictors from T1 and T2 are given in Table 2 and Figure 4A–G. The algorithms predicted the final measurement at T3 with accuracies between 96.60% and 98.34%. The lasso and elastic net methods revealed excellent correlations (ICCs ≥ 0.9), while the least squares method was found to have moderate correlation between the predicted and actual values (0.5 < ICCs < 0.75). All remaining methods revealed a good correlation between the predicted and actual values (0.75 < ICCs < 0.90). The top correlating coefficients picked up by the methods were found to be the Y axis of growth at T2 followed by the Y axis of growth at T1, lower face height at T2, and mandibular plane angle at T2 (Figure 4H–K). Supplemental Figure S3 shows the correlation matrix with heatmap, representing the correlation between the variables used to predict the Y axis of growth at T3 using variables from T1 and T2 combined.

**Figure 5:**
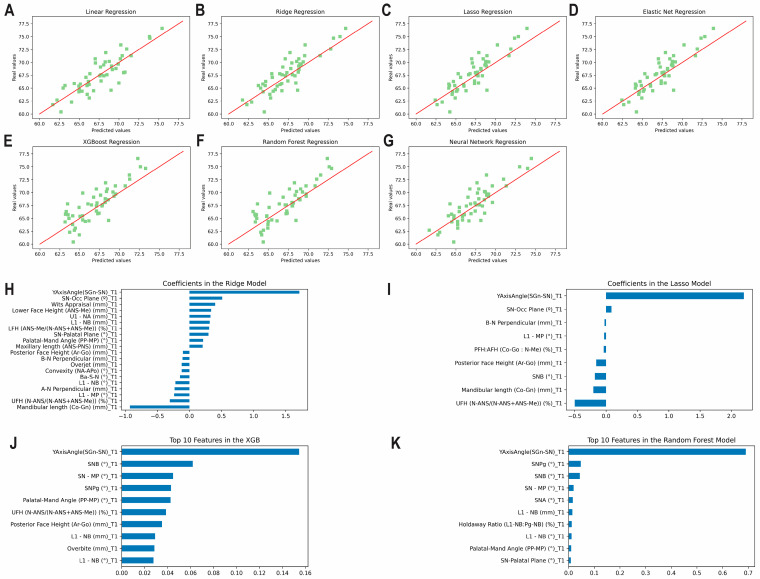
Predicting the Y axis of growth at T3 using variables from T1 alone. Scatter plots for the predicted and actual values with lines of best fit for the: (**A**) Least Squares (linear); (**B**) Ridge; (**C**) Lasso; (**D**) Elastic Net; (**E**) XGBoost; (**F**) Random Forest; (**G**) Neural Network models. Top correlating coefficients for the (**H**) Ridge model; (**I**) Lasso model; (**J**) XGBoost model; (**K**) Random Forest model.

The results of the prediction analysis of the Y axis of growth using predictors from T1 alone are given in Table 2 and Figure 5A–G. Accuracies between 97.52% and 97.89% were observed amongst the seven models. The ridge method was found to be the most accurate (MAE: 1.42°). All methods revealed a good correlation between predicted and actual values (0.75 < ICCs < 0.90). The top correlating coefficient was the Y axis of growth at T1, followed by occlusal plane angle at T1, SNB angle at T1, and mandibular plane angle at T1 (Figure 5H–K). Supplemental Figure S4 shows the correlation matrix with heatmap, representing the correlation between the variables used to predict the Y axis of growth at T3 using variables from T1 only.

### 3.5. Overfitting

Overfitting is a situation where a model is too complex and is trained on a limited set of data so that it becomes overly specific to the training data and fails to generalize well to new, unseen data. One way to alleviate the risk of overfitting was to decrease the number of input features to the clinically most relevant ones. For this purpose, a second round of experiments were performed by using only the variables selected by the Lasso model at the first round. The selected variables used to predict the mandibular length at T3 were mandibular length at T1 and T2, maxillary length at T1, lower face height at T1, Ba-S-N at T2, upper face height at T1, B-N perpendicular at T2, Wits appraisal at T1, and age at T2. The selected variables used to predict the Y axis of growth at T3 were Y axis of growth at T1 and T2, mandibular length at T1 and T2, L1-MP at T2, upper face height/total face height ratio at T2, and posterior face height at T2. The results of the prediction analysis of mandibular length and Y axis of growth at T3 by using selected variables are shown in Table 3. In prediction of mandibular length at T3, the MAEs of the algorithms ranged between 3.67 and 4.69 mm. The accuracy of the least squares model (97.18%) significantly improved by using only the selected variables for training. On the other hand, accuracies of the XGBoost and random forest models showed a slight decrease (96.40% and 96.76%, respectively). Other models showed only minor changes in their accuracies. In prediction of Y axis of growth, the MAEs of the algorithms ranged between 0.99 and 1.24. The greatest improvement in accuracy was observed with the least squares model (from 96.60% to 98.50%). No significant changes were found with the other models. Overall, the results showed that the least squares model benefitted the most from reducing the number of input features, while only minor changes in accuracies were noted with the other models.

### 3.6. Method Comparison (ANOVA)

While predicting the mandibular length at T3, there was no significant interaction between methods and time points used for predictors (*p* = 0.235). No significant differences were found among methods (*p* = 0.904). There was no significant difference between using predictors from T1 alone and using predictors from T1 and T2 together (*p* = 0.209).

While predicting the Y axis of growth at T3, there was no significant interaction between methods and time points used for predictors (*p* = 0.549). However, using predictors from T1 alone resulted in a significantly larger difference between predicted and actual measurements than using predictors from T1 and T2 together (*p* = 0.007), where using only T1 predictors underestimated the measurements by a larger amount on average than the combination of T1 and T2 predictors. The least squares prediction had a significantly larger difference between predicted and actual measurements than all other prediction methods (*p* ≤ 0.001), where least squares overestimated the measurements. The XGBoost prediction had a significantly larger difference between predicted and actual measurements than neural net (*p* = 0.022) and ridge (*p* = 0.027), where XGBoost underestimated the measurements by a larger amount on average than the other two methods.

## 4. Discussion

A significant amount of variability in the amount and direction of the pubertal mandibular growth exists among genders, races and even between the individuals having the same age and gender. Therefore, to analyze the complex growth pattern of the mandible, more specific samples in terms of gender and age were employed. Only the records from boys at the circumpubertal stage were analyzed in this study. Using records of children from time points of 11 to 16 years allowed us to investigate peak growth and maturation for most males, ending as peak growth begins to level for a more stable estimate of final mandibular position after growth. In addition to a specified gender and age interval, only individuals with no significant skeletal malocclusions were included because mandibular growth pattern significantly differs in Class II [29] and Class III malocclusions [30]. By doing so, we aimed to create a norm reference for the pubertal mandibular growth in individuals without any jaw discrepancies. This will build a base algorithm for future AI models to build off that will include other Angle’s classifications.

In this study, a total of 39 independent input variables were used to predict the two dependent outcome variables. Leslie et al. [5] noted that the stepwise regression used in past studies [4] allows overfitting of models and is only exploratory when so many independent variables are in play. Skieller et al. [4] even noted that it is practically impossible to investigate all combinations with such large numbers of independent variables. However, the use of AI alleviated this concern. The findings of this study demonstrated that, although too many variables were used as input data, certain features were picked up by the algorithms regardless of the methods used. Among others, mandibular length and the Y axis of growth at previous time points were seen to be the most important predictors for the same measurements at T3, but other measurements also served a role as important predictors for the outcome. This was an interesting result, as Björk [3] determined that final mandibular length could not be judged from its size before puberty. Depending on the utilized algorithm and the number of timepoints used to predict mandibular length, maxillary length, lower face height, upper face height, and posterior face height, among others were found to be important predictive factors as inputs for the algorithm. Similarly, when predicting the Y axis of growth, SNB, SN-Occl plane, the Wits appraisal, SNPg, Palatal-Mandibular plane angle, SN-MP, and lower face height, which have been seen in previous literature [31], were seen along with a few other predictive variables. However, as with mandibular length, their importance decreased by over half relative to the leading predictors. It stands to reason that with mandibular length being an antero-posterior (AP) measurement, the top predictive factors will be mandibular length and maxillary length as these are also AP measurements. It is interesting to note that the majority of other predictive factors were vertical measurements. Similarly, when predicting the Y axis of growth as a prediction of direction (forward or vertical growth), the predictors were all related to AP and vertical positioning.

None of the ML techniques used in this study showed a clear superiority to others in predicting the post-pubertal mandibular length. However, slight differences between the techniques were observed in predicting the Y axis of growth, where least squares resulted in a significantly larger difference between predicted and actual measurements than all other prediction methods. The reason for the differences was found to be that the least squares method overestimated the measurements. From the results, it was clear that the least squares approach was overfitting data slightly compared to the ridge and lasso models. Considering the small sample size and relatively large number of variables, this comes as no surprise. However, when the analyses were repeated with only the selected variables, the accuracy of the least squares model significantly improved. The overfitting effect was already mitigated in the ridge and lasso models by penalizing model coefficients. The ridge regression uses the L2 norm whereas lasso uses L1 norm to regularize model coefficients. Thanks to the L1 norm regularizer, lasso shrinks some of the model coefficients to zero leading to a sparse solution, where only a subset of the model coefficients is assigned nonzero values. When overall performances were considered, the authors would recommend the lasso model for further studies, as this technique was consistently observed to be either the 1st or 2nd most accurate technique when considering the MAE.

No statistically significant difference was seen between using records from T1 alone or T1 combined with T2, when predicting the post-pubertal mandibular length. This is very promising as the ideal clinical scenario would include the ability to predict mandibular growth from a single time point taken earlier in a patient’s development. Though there was a significant difference when predicting the Y axis of growth, there was still good accuracy and correlation when using T1 alone. In future studies, it would be prudent to continue to monitor the accuracy of using T1 alone vs using T1 combined with T2.

The authors acknowledge that the current study contained some limitations, some of which were present in other growth prediction studies, too. The first was the acquired sample size, particularly when training the ML methods. While a power analysis was conducted and the projected number of subjects were identified, a larger sample size might have led to more accurate and significant results from the algorithms developed. These sample sizes often are in the realm of thousands or sometimes even millions of “subjects”. Likewise, more training sets provide a better functioning algorithm. Many images had cut off soft tissue that may have been used as more predictive input and varying levels of clarity among radiographs. Finally, though excellent intra-rater reliability was obtained in the study, landmark detection and human tracing errors always have the potential to introduce bias into any study involving cephalometric analysis. Other studies have used S-Gn rather than Co-Gn when assessing mandibular length [7]. This may be of better use in future studies as Co is often more difficult to locate. Using quantitative data has more risks of introducing bias and error: in the future it would be best to use image pattern analysis.

## 5. Conclusions

All tested ML algorithms accurately predicted the post-pubertal mandibular length and Y axis of growth. The best predictors for the post-pubertal mandibular length were mandibular and maxillary lengths, and lower face heights at the earlier timepoints. The best predictors for the post-pubertal Y axis of growth included the Y axis of growth, lower face height, and mandibular plane angle at the earlier timepoints. No significant difference was found among the accuracies of the ML techniques tested, except for least squares, which had a significantly larger error than all other prediction methods in predicting the Y axis of growth. However, the accuracy of the least squares model was significantly improved by reducing the number of input variables. Additional research with larger sample sizes and more time points will be necessary to obtain more accurate and generalizable predictions.

## Figures and Tables

**Table 1:** The results of the prediction analysis of mandibular length at T3.

	Using Variables from T1 and T2					Using Variables from T1				
Method	ME (SD)	MAE	RMSE	ICC	Accuracy %	ME (SD)	MAE	RMSE	ICC	Accuracy %
Least Squares	−1.32 (0.99)	5.47	6.95	0.76	95.80	−0.22 (0.77)	3.90	5.35	0.80	97.00
Ridge	−0.37 (0.74)	3.45	5.17	0.80	97.35	−0.16 (0.82)	3.99	5.69	0.72	96.94
Lasso	−0.66 (0.70)	3.31	4.91	0.82	97.46	−0.34 (0.79)	3.67	5.49	0.75	97.18
Elastic Net	−0.57 (0.72)	3.35	5.05	0.81	97.43	−0.36 (0.81)	3.96	5.61	0.74	96.96
XGBoost	−0.48 (0.75)	3.78	5.20	0.82	97.10	−0.72 (0.73)	3.52	5.11	0.82	97.30
Random Forest	−1.03 (0.72)	3.07	5.11	0.81	97.64	−0.35 (0.77)	3.51	5.32	0.79	97.30
Neural Net	−0.80 (0.72)	4.10	5.08	0.84	96.85	−0.20 (0.72)	3.47	4.99	0.82	97.33

ME: Mean error, SD: Standard deviation, MAE: Mean absolute error, RMSE: Root mean square error, ICC: Intraclass correlation coefficient.

**Table 2:** The results of the prediction analysis of Y axis of growth at T3.

	Using Variables from T1 and T2					Using Variables from T1				
Method	ME (SD)	MAE	RMSE	ICC	Accuracy %	ME (SD)	MAE	RMSE	ICC	Accuracy %
Least Squares	0.52 (0.41)	2.29	2.91	0.68	96.60	−0.09 (0.26)	1.45	1.82	0.85	97.85
Ridge	−0.15 (0.23)	1.29	1.61	0.87	98.09	−0.38 (0.25)	1.42	1.77	0.85	97.89
Lasso	−0.17 (0.21)	1.12	1.43	0.90	98.34	−0.51 (0.25)	1.43	1.79	0.84	97.88
Elastic Net	−0.17 (0.21)	1.12	1.43	0.90	98.34	−0.51 (0.25)	1.43	1.79	0.84	97.88
XGBoost	−0.46 (0.24)	1.35	1.74	0.85	98.00	−0.70 (0.28)	1.67	2.08	0.78	97.52
Random Forest	−0.41 (0.25)	1.38	1.75	0.85	97.95	−0.62 (0.28)	1.64	2.03	0.80	97.57
Neural Net	−0.05 (0.23)	1.24	1.59	0.88	98.16	−0.45 (0.27)	1.49	1.90	0.82	97.79

ME: Mean error, SD: Standard deviation, MAE: Mean absolute error, RMSE: Root mean square error, ICC: Intraclass correlation coefficient.

**Table 3:** The results of the prediction analysis of mandibular length and Y axis of growth at T3 by using selected variables from T1 and T2.

	Mandibular Length				Y Axis of Growth			
Method	ME	MAE	RMSE	Accuracy %	ME	MAE	RMSE	Accuracy %
Least Squares	0.69	3.67	5.39	97.18	−0.11	1.01	1.32	98.50
Ridge	1.11	3.77	5.37	97.10	−0.11	1.01	1.32	98.50
Lasso	1.18	3.88	5.91	97.02	−0.23	1.00	1.34	98.52
Elastic Net	1.22	3.78	5.53	97.10	−0.20	0.99	1.32	98.53
XGBoost	2.79	4.69	7.31	96.40	0.05	1.24	1.58	98.16
Random Forest	1.86	4.22	6.74	96.76	−0.39	1.22	1.58	98.19
Neural Net	1.50	4.25	6.64	96.74	−0.15	1.04	1.37	98.46

ME: Mean error, MAE: Mean absolute error, RMSE: Root mean square error.

## Data Availability

The data underlying this article are available in the article. The datasets were derived from sources in the public domain from the AAOF Legacy Collection at https://www.aaoflegacycollection.org (accessed on 25 April 2023).

## Supplementary Materials

The following supporting information can be downloaded at https://www.mdpi.com/article/10.3390/diagnostics13091553/s1. Figure S1: Correlation matrix with heatmap, representing the correlation between the predictors from T1 and T2 together used to predict mandibular length at T3; Figure S2: Correlation matrix with heatmap, representing the correlation between the predictors from T1 alone used to predict mandibular length at T3; Figure S3: Correlation matrix with heatmap, representing the correlation between the predictors from T1 and T2 together used to predict the Y axis of growth at T3; Figure S4: Correlation matrix with heatmap, representing the correlation between the predictors from T1 alone used to predict the Y axis of growth at T3; Table S1: Description of the cephalometric measurements; Table S2: Intra-examiner repeatability of the cephalometric measurements; Table S3: Descriptive statistics of the cephalometric variables at T1, T2 and T3; Table S4: List of abbreviations.
