# Supplementary figures and images for: Prediction of the Post-Pubertal Mandibular Length and Y Axis of Growth by Using Various Machine Learning Techniques: A Retrospective Longitudinal Study
Source: Diagnostics (Basel). 2023 Apr 26;13(9):1553. doi: 10.3390/diagnostics13091553 (PMC10178146; doi:10.3390/diagnostics13091553)

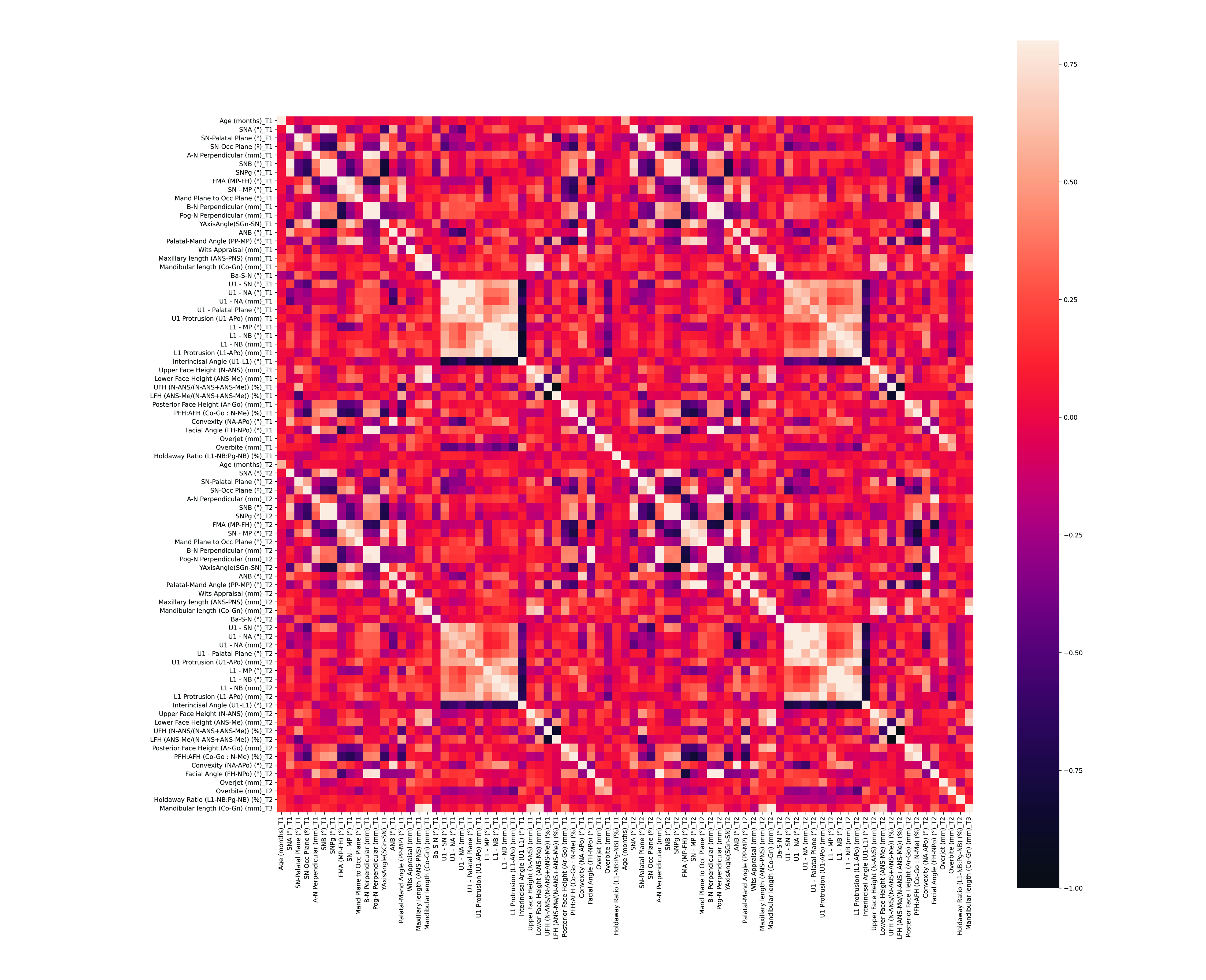

Supplement: Supplementary file 1 [file diagnostics-13-01553-s001.zip › Supp_Figure 1.tif]

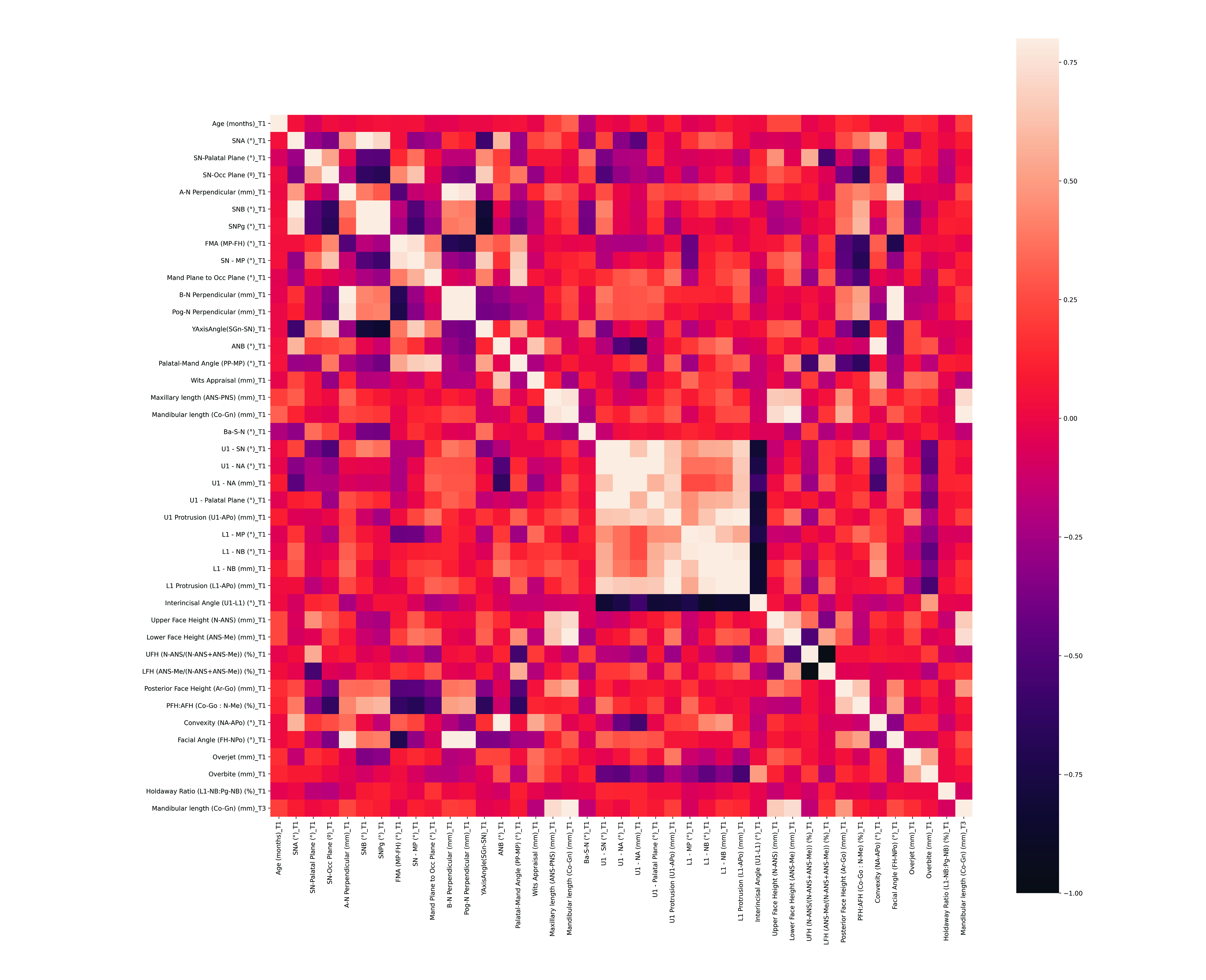

Supplement: Supplementary file 1 [file diagnostics-13-01553-s001.zip › Supp_Figure 2.tif]

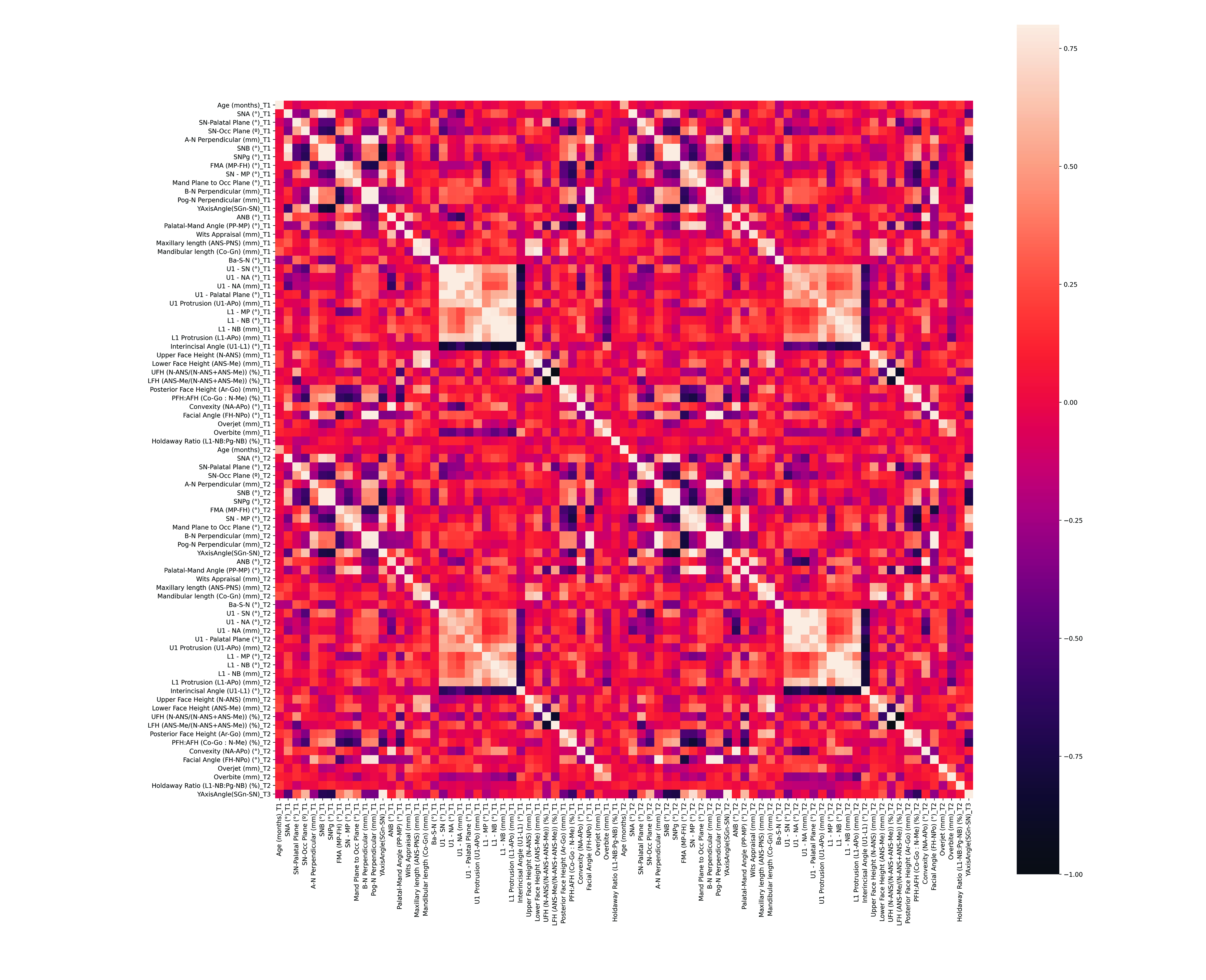

Supplement: Supplementary file 1 [file diagnostics-13-01553-s001.zip › Supp_Figure 3.tif]

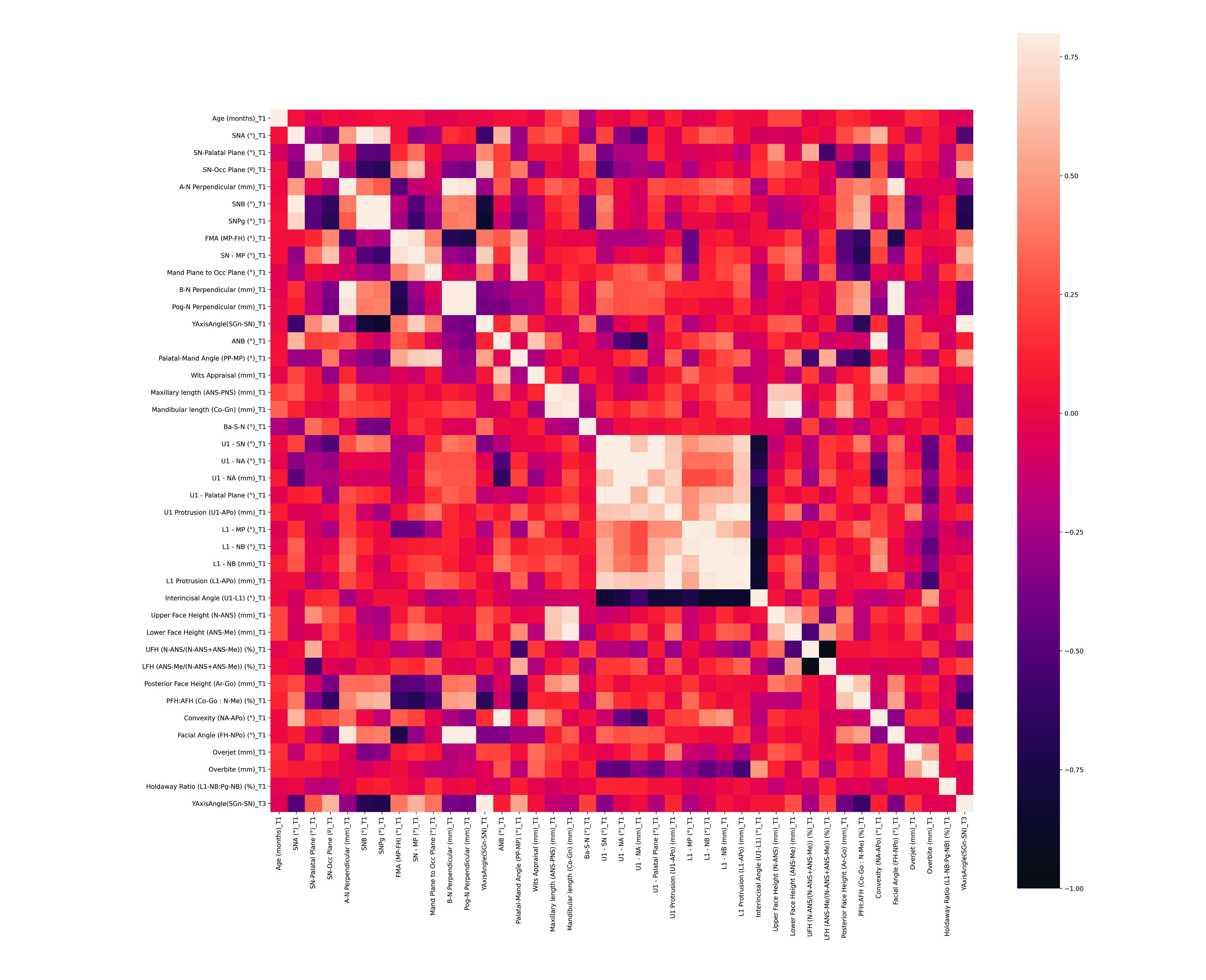

Supplement: Supplementary file 1 [file diagnostics-13-01553-s001.zip › Supp_Figure 4.tif]
